# FCHSD1 and FCHSD2 Are Expressed in Hair Cell Stereocilia and Cuticular Plate and Regulate Actin Polymerization *In Vitro*

**DOI:** 10.1371/journal.pone.0056516

**Published:** 2013-02-20

**Authors:** Huiren Cao, Xiaolei Yin, Yujie Cao, Yecheng Jin, Shan Wang, Yanhui Kong, Yuexing Chen, Jiangang Gao, Stefan Heller, Zhigang Xu

**Affiliations:** 1 Shandong Provincial Key Laboratory of Animal Cells and Developmental Biology, Institute of Developmental Biology, School of Life Sciences, Shandong University, Jinan, Shandong, People’s Republic of China; 2 Departments of Otolaryngology – Head & Neck Surgery and Molecular & Cellular Physiology, Stanford University School of Medicine, Stanford, California, United States of America; University of Oldenburg, Germany

## Abstract

Mammalian FCHSD1 and FCHSD2 are homologous proteins containing an amino-terminal F-BAR domain and two SH3 domains near their carboxyl-termini. We report here that FCHSD1 and FCHSD2 are expressed in mouse cochlear sensory hair cells. FCHSD1 mainly localizes to the cuticular plate, whereas FCHSD2 mainly localizes along the stereocilia in a punctuate pattern. Nervous Wreck (Nwk), the *Drosophila* ortholog of FCHSD1 and FCHSD2, has been shown to bind Wsp and play an important role in F-actin assembly. We show that, like its *Drosophila* counterpart, FCHSD2 interacts with WASP and N-WASP, the mammalian orthologs of *Drosophila* Wsp, and stimulates F-actin assembly *in vitro*. In contrast, FCHSD1 doesn’t bind WASP or N-WASP, and can’t stimulate F-actin assembly when tested *in vitro*. We found, however, that FCHSD1 binds via its F-BAR domain to the SH3 domain of Sorting Nexin 9 (SNX9), a well characterized BAR protein that has been shown to promote WASP-Arp2/3-dependent F-actin polymerization. FCHSD1 greatly enhances SNX9’s WASP-Arp2/3-dependent F-actin polymerization activity. In hair cells, SNX9 was detected in the cuticular plate, where it colocalizes with FCHSD1. Our results suggest that FCHSD1 and FCHSD2 could modulate F-actin assembly or maintenance in hair cell stereocilia and cuticular plate.

## Introduction

Inner ear hair cells are auditory and vestibular receptor cells, responsible for converting mechanical stimuli into electrical signals in a process called mechanoelectrical transduction. They feature F-actin-based, microvilli-like protrusions on their apical surface, named stereocilia, which are organized into several rows of increasing heights. Mechanoelectrical transduction happens near the tips of middle and short rows of stereocilia [1]. In each stereocilium, several hundred cross-linked parallel actin filaments form a rigid paracrystalline array. The stereocilia have rootlets that are inserted into a dense filamentous actin network, the cuticular plate, which occupies the apical cell cytoplasm and serves as an anchor for stereocilia [2], [3]. There are several types of extracellular links among stereocilia, including tip-links, top-connectors, lateral-links, ankle-links, and kinociliary-links. By connecting stereocilia with each other and with the microtubule-based kinocilium, these links play important roles in maintaining stereociliary integrity and stability and some of them are directly involved in the transduction process [4], [5].

Stereociliary F-actin is polarized, with the barbed (plus) ends at the tips and the pointed (minus) ends at the base. During development, stereociliary F-actin experiences turnover. In a process called tread-milling, actin monomers join the barbed end of the filament and depart the filament from the pointed end [6], [7]. Recently, Zhang and colleagues measured the turnover of stereociliary protein using multi-isotope mass spectrometry (MIMS) imaging and found that mature stereocilia are remarkably stable; only the stereociliary tips displayed turnover and tread-milling was not observed [8]. Several proteins have been described at different locations in stereocilia, and are suggested to play important roles in formation, maintenance and function of this highly organized organelle. For example, Myosin 3, Espin, Myosin 15a and Whirlin localize toward the tips of stereocilia where they might be involved in stereociliary elongation or actin turnover [9], [10], [11], [12], [13]. Twinfilin 2, on the other hand, is only present at the tips of middle and short rows of stereocilia; Twinfilin 2 is able to suppress the elongation of stereociliary actin filaments and was put forward as potential elongation regulator during postnatal hair bundle maturation [14], [15]. Taperin, a protein with partial homology to the actin-caping protein phostensin, localizes at the taper region of stereocilia, and was suggested to modulate actin dynamics through direct or indirect interaction with pointed ends of actin filaments [16], [17]. The cuticular plate consists of a network of actin filaments, and its development and maintenance are less understood.

Because F-actin dynamics in stereocilia and cuticular plate appear to be highly controlled, we hypothesized that additional actin regulators might play roles in these unique organelles. We focused on FCH (FER-CIP4 homology) domain and double SH3 domains containing proteins 1 and 2 (FCHSD1 and FCHSD2), which are mammalian orthologs of *Drosophila* Nervous Wreck (Nwk) [18]. FCHSD1, FCHSD2 and Nwk belong to the F-BAR (FCH and Bin-Amphiphysin-Rvs) protein family. BAR/F-BAR proteins are a class of cytosolic proteins that have the ability to bind plasma membranes and thereby to couple other proteins such as actin cytoskeleton with plasma membranes [19], [20]. They all have a BAR or F-BAR domain at the amino-terminus and other domains such as SH3, RhoGAP, or PH domains near the carboxyl-terminus, which may mediate the interaction with actin regulatory proteins. Functionally, BAR/F-BAR proteins have been shown to interact with plasma membranes and are involved in F-actin modulation, which implicates them in many fundamental biological processes, such as endocytosis, exocytosis, cytoskeletal reorganization, and cell migration [21]. Nwk, the ortholog of FCHSD1 and FCHSD2, was first identified in *Drosophila* where mutations of *nwk* gene cause excessive growth of neuromuscular junctions (NMJ), resulting in seizure-like spasms and paralysis [22]. Nwk modulates F-actin dynamics or endocytosis via binding to F-actin regulatory proteins Wsp, Dynamin, Dap160, and Snx16 [23], [24], [25]. At present the function of FCHSD1 and FCHSD2 is largely unknown, except that FCHSD2, which was named Carom when first identified, was shown to bind scaffolding proteins MAGI-1 and CASK and associate with cytoskeleton [26].

In this study, we demonstrate that FCHSD1 localizes to the cuticular plate, whereas FCHSD2 mainly localizes along the stereocilia. Similar to their fly ortholog, both proteins regulate F-actin assembly. FCHSD2 binds WASP and N-WASP, and stimulates WASP-Arp2/3-mediated F-actin polymerization *in vitro*. FCHSD1 doesn’t bind WASP or N-WASP, and has no effect on WASP-Arp2/3-mediated F-actin polymerization by itself. Instead, it interacts with Sorting Nexin 9 (SNX9), a well characterized BAR protein involved in actin cytoskeleton regulation [27], and enhances SNX9’s WASP-Arp2/3-dependent F-actin polymerization activity. Interestingly, in hair cells, SNX9 is present in the cuticular plate, where it colocalizes with FCHSD1. Our findings suggest that FCHSD1 and FCHSD2 are F-actin regulatory proteins expressed in stereocilia and cuticular plate of sensory hair cells and may be involved in development and/or maintenance of the F-actin cytoskeleton of these organelles.

## Methods

### Ethics Statement

All animal experiments were approved by the Ethics Committee of Shandong University (Permit Number: ECAESDUSM 20123004) and conducted accordingly.

### DNA Constructs and Antibodies

Mouse FCHSD1, FCHSD2 and SNX9 cDNAs were amplified from mouse organ of Corti cDNA and cloned into expression vectors pcDNA3.1(+) or pEGFP-C2. The cDNAs encoding FCHSD1-F-BAR (1–315aa), FCHSD1ΔF-BAR (302–688aa), SNX9-SH3 (1–65aa), SNX9-LC (61–200aa), SNX9-PX-BAR (200–595aa) were subcloned into pEGFP-C2 or pEGFP-N2. WASP and N-WASP cDNAs were gifts from Dr. Michael Rosen (UT Southwestern Medical Center) and were cloned into pcDNA3.1(+). Mouse monoclonal anti-FCHSD1 antibody was purchased from Novus (Cat. No. H00089848-M01). Mouse polyclonal anti-FCHSD1 antibody was purchased from Abcam (Cat. No. ab67017). Rabbit polyclonal anti-FCHSD2 antibody was a gift from Yutaka Hata (Tokyo Medical and Dental University). Rabbit polyclonal anti-SNX9 antibody was purchased from Proteintech (Cat. No. 15721-1-AP).

### Extraction of RNA and RT-PCR

Total RNA from postnatal day 5 mouse (C57BL/6) tissues was extracted using RNeasy Micro Kits (Qiagen, Valencia, CA) according to the manufacturer’s protocol. Reverse transcription was carried out at 42°C for 1 hour in a 25 µL reaction mixture that contained 1 µg of total RNA, 10 pmol of oligo-dT, and 200 units of Super-Script III reverse transcriptase (Invitrogen, Carlsbad, CA). Polymerase chain reaction was performed using this cDNA as template with the following primers: *Fchsd1* forward primer, AGGGGCCTGGAGTCTTTTC, reverse primer, GGCGGCCCCTTTCACCT (311 bp); *Fchsd2* forward primer, TGTTCATCAGCAGCGGGTTCTAAA, reverse primer, AAGGGCTGGAGCTGCTGTCATCAA (365 bp); *β-actin* forward primer, ACGGCCAGGTCATCACTATTG, reverse primer, AGGGGCCGGACTCATCGTA (372 bp). To achieve the best possible sensitivity and specificity, cycle lengths for different PCR reaction sets were adjusted between 30 and 33 cycles, and annealing temperatures were adjusted between 58 and 60°C. The PCR products were separated by electrophoresis on agarose gel.

### Western Blot and Co-Immunoprecipitation

Tissues of postnatal day 5 mouse (C57BL/6) were dissected and homogenized in ice-cold lysis buffer consisting of 150 mM NaCl, 50 mM Tris at pH 7.5, 1% (vol/vol) Triton X-100, 1 mM PMSF, and 1 X protease inhibitor cocktail (Sigma-Aldrich, Saint Louis, MO). After centrifuging at 4°C, the supernatant was collected and separated by polyacrylamide gel electrophoresis, then transferred to PVDF membrane and detected with corresponding antibodies. For Co-Immunoprecipitation, HEK293 cells were transfected with expression vectors using Lipofectamine 2000 (Invitrogen), then washed with PBS 24–48 hours after transfection and lysed in ice-cold lysis buffer consisting of 150 mM NaCl, 50 mM Tris at pH 7.5, 1% (vol/vol) Triton X-100, 1 mM PMSF, and 1 X protease inhibitor cocktail (Sigma-Aldrich) or RIPA buffer containing PMSF and protease inhibitor cocktail. After centrifuging at 4°C, the supernatant was collected and incubated with anti-Myc antibody (Sigma) and Protein A agarose (GE)/Protein G agarose (Invitrogen) over night at 4°C. Immunoprecipitated proteins were washed five times with lysis buffer and separated by polyacrylamide gel electrophoresis, then transferred to PVDF membrane and detected with corresponding antibodies.

### Whole-Mount Immunostaining

All steps were performed at room temperature unless otherwise indicated. Samples of organ of Corti from Balb/c or C57BL/6 mice were dissected and fixed with 4% paraformaldehyde, then permeabilized and blocked with PBT1 buffer (0.1% Triton X-100, 1% BSA, 5% heat-inactivated goat serum in PBS, pH 7.3) for 30 minutes. Samples were incubated overnight at 4°C with 10 µg/ml anti-FCHSD1 or anti-FCHSD2 antibody (or 3 µg/ml anti-SNX9 antibody) diluted in PBT1, then washed twice with PBT1 for 10 minutes and twice with PBT2 (0.1% Triton X-100, 0.1% BSA in PBS) for 5 minutes, and incubated with 7.5 µg/ml FITC (or Cy5)-conjugated secondary antibody (Jackson ImmunoResearch Inc., West Grove, PA) in PBT2 for 1 hour, followed by washing with PBT2 twice for 10 minutes and PBS once for 10 minutes. Samples were incubated with 4 µg/ml TRITC-conjugated phalloidin in PBS for 30 minutes followed by three 10-minutes washes with PBS, then mounted in Cytomation Fluorescent Mounting Medium (Dako, Carpinteria, CA) and imaged with a confocal microscope (LSM Pascal, Zeiss, Germany).

### Yeast Two-Hybrid Screen

The screen was performed as described previously [28]. Chicken FCHSD1 cDNA was amplified from chicken basilar papilla cDNA and cloned into vector pBD-GAL4 Cam (Stratagene) to express the bait protein. The yeast strain AH109 (Clontech, Mountain View, CA) transformed with this bait plasmid was then transformed with a chicken basilar papilla cDNA library in the HybriZAP two-hybrid vector [29]. *HIS3* was used as the primary reporter gene for the screen in the presence of 7.5 mM of 3-amino-1,2,4-triazole. Totally 6.3×10^6^ transformants were selectively screened and positive colonies were tested for activation of two other reporter genes *ADE2* and *lacZ*. The pAD-GAL4-based phagemid vectors in triple-positive yeast colonies were recovered and cDNA inserts were sequenced.

### Protein Purification

The cDNAs encoding FCHSD1ΔC (amino acids 1–627), FCHSD2ΔC (amino acids 1–631), and full-length SNX9 were amplified by PCR and cloned into pGEX-4T-2. The cDNA encoding WASP145 (missing amino-terminal 145 amino acids) was amplified by PCR and cloned into pET-28a. The plasmids were then transformed into E. coli BL21 (DE3) strain to express fusion proteins in the presence of isopropyl-b-D-thiogalactopyranoside (IPTG) (1 mM, 0.1 mM, 0.05 mM, 1 mM for GST-FCHSD1ΔC, GST-FCHSD2ΔC, GST-SNX9 and His-WASP145, respectively) over night at room temperature. The fusion proteins were purified using Glutathione Sepharose 4B (GE Healthcare) or Ni-NTA agarose (TransGen, Beijing, China) according to manufacturer’s instructions and further fractionated by gel filtration chromatography (Superdex-200, Pharmacia, for GST-FCHSD1ΔC, GST-FCHSD2ΔC and GST-SNX9) or anion-exchange (Source-15Q, Pharmacia, for His-WASP145). Bovine Arp2/3 complex was purchased from Cytoskeleton, Inc (Denver, CO). Actin was isolated from rabbit skeletal muscle as described previously [30] and G-actin was further purified using Sephacryl S-300 chromatography at 4°C in Buffer G (0.2 mM ATP, 0.1 mM CaCl_2_, 0.5 mM DTT, 0.1 mM imidazole, and 5 mM Tris-HCl, pH 8.0) [31]. To trace the dynamics of actin polymerization, actin was labeled on Cys-374 with pyrene iodoacetamide [31].

### Actin Polymerization Assay

Monomeric actin (3 µM, 10% pyrene-labeled) was incubated with different protein samples (Arp2/3 protein complex, 20 nM; His-WASP145, 40 nM; GST-FCHSD1ΔC, 1 μΜ; GST-FCHSD2ΔC, 1 μΜ; GST-SNX9, 1 μΜ; GST, 1 μΜ) for 4 minutes at room temperature. Then 1/10 volume of 10×KMEI (500 mM KCl, 10 mM MgCl_2_, 10 mM EGTA, and 100 mM imidazole-HCl, pH 7.0 ) was added into the mixture and F-actin polymerization was monitored with a QuantaMaster Luminescence QM 3 PH fluorometer (Photon Technology International, South Brunswick, NJ) using an excitation wavelength of 365 nm and an emission wavelength of 407 nm.

## Results

### Mammalian FCHSD1 and FCHSD2 are Widely Expressed

**Figure 1:**
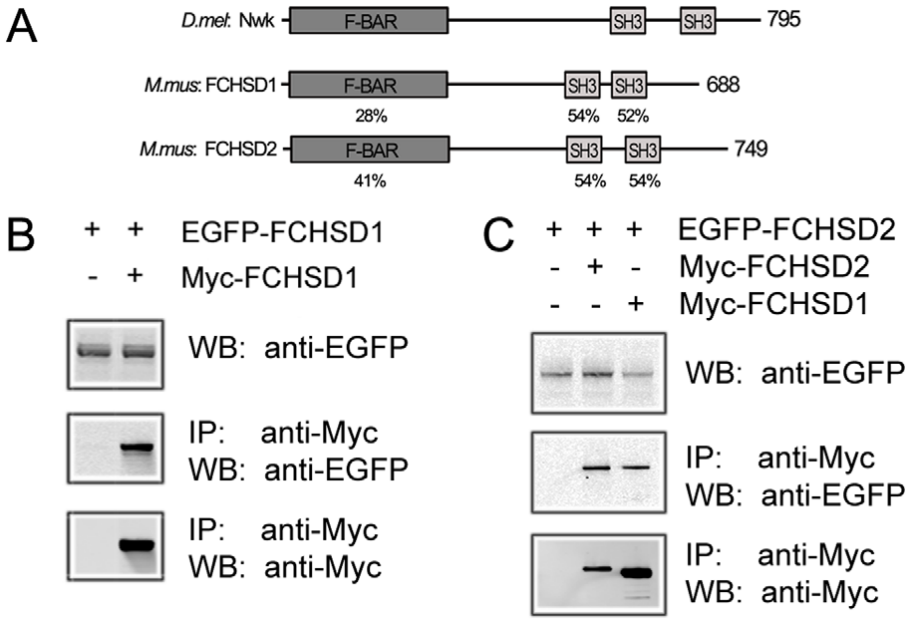
Mammalian FCHSD1 and FCHSD2 are homologous to fly Nwk and form oligmers with each other. (A) Schematic drawing of the domain structures of *Mus musculus* FCHSD1, FCHSD2 and *Drosophila melanogaster* Nwk. The identity of F-BAR domains and SH3 domains between FCHSD1, FCHSD2 and Nwk is indicated below each domain. (B and C) Western blots showing that FCHSD1 and FCHSD2 were co-immunoprecipitated with themselves and with each other. Expression vectors were transfected into HEK293 cells to express epitope-tagged FCHSD1 and FCHSD2, and cell lysates were subjected to immunoprecipitation. IP indicates antibody used for immunoprecipitation and WB indicates antibody used for detection.

FCHSD1 and FCHSD2 share similar domain organization with their fly ortholog Nwk, containing an amino-terminal F-BAR domain and two SH3 domains near their carboxyl-termini (Fig. 1A). Their F-BAR and SH3 domains are well conserved between fly and mouse, with over 40% amino-acid identity for the F-BAR domains and over 50% amino-acid identity for the SH3 domains. The only exception is FCHSD1’s F-BAR domain, which has only 28% identity with Nwk’s F-BAR domain. We amplified the cDNA of mouse FCHSD1 and FCHSD2 from a cochlear cDNA library, subcloned the coding sequences into expression vectors and expressed epitope-tagged full-length proteins in cultured cells. Both epitope-tagged proteins showed an exclusive cytoplasmic localization in transfected HEK293 cells (Data not shown). It has been shown that F-BAR proteins can form dimers [32], so we performed co-immunoprecipitation (co-IP) experiments to examine whether FCHSD1 and FCHSD2 also do so. Our results show that FCHSD1 and FCHSD2 could form homo- and hetero-oligomers (Fig. 1B and 1C).

**Figure 2:**
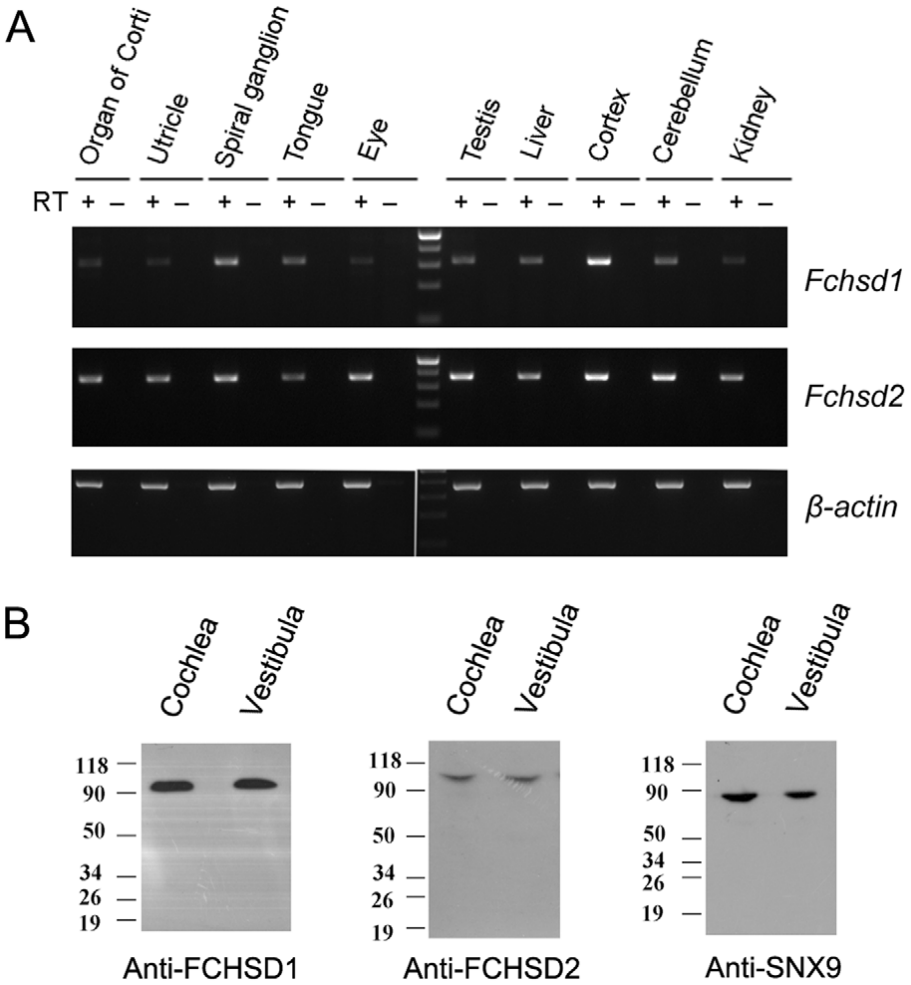
Expression analysis of mouse Fchsd1 and Fchsd2 in different tissues. (A) Total RNA from postnatal day 5 mouse tissues was extracted and used as template for reverse transcription. PCR was performed using this cDNA as template. Upper panel, *Fchsd1* mRNA is expressed more abundantly in nervous tissues. Middle panel, *Fchsd2* mRNA is expressed ubiquitously in all tissues examined. Lower panel, *β-actin* specific primers were used as the RT-PCR template control. (B) Total proteins of postnatal day 5 mouse cochlea and vestibula were extracted and separated by PAGE and detected with antibodies against FCHSD1(Novus), FCHSD2, or SNX9.

To examine the expression of mouse *Fchsd1* and *Fchsd2* mRNAs in different tissues, we performed RT-PCR experiments. Our results reveal that both genes are detected in all the organs and tissues examined. *Fchsd1* appeared to be most abundantly expressed in the cortex, whereas *Fchsd2* appeared generally expressed at easily detectable levels (Fig. 2A).

### FCHSD1 and FCHSD2 Are Detectable in Stereocilia and Cuticular Plate

**Figure 3:**
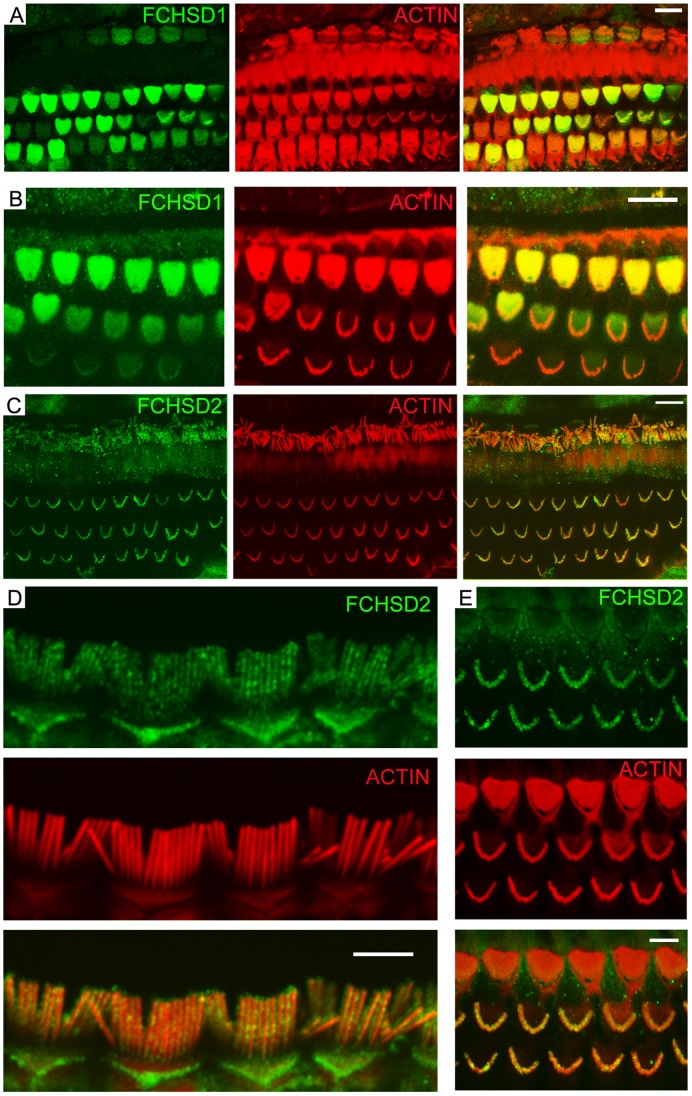
FCHSD1 and FCHSD2 immunolocalization in mouse cochlear hair cells. Shown are single confocal sections. FCHSD1 or FCHSD2 immunoreactivity visualized with Cy5 or FITC-conjugated secondary antibody was distinctly associated with stereocilia or cuticular plate, which were visualized with rhodamine-conjugated phalloidin. (A) FCHSD1 immunoreactivity in the cuticular plate of 3-week old mouse cochlear hair cells. (B) FCHSD1 immunoreactivity in the cuticular plate of 3-week old mouse outer hair cells. (C) FCHSD2 immunoreactivity in the hair bundles of 6-week old mouse cochlear hair cells. (D) FCHSD2 immunoreactivity in the hair bundles of 7-month old mouse inner hair cells. (E) FCHSD2 immunoreactivity in the hair bundles of 2-week old mouse outer hair cells. The Novus FCHSD1 antibody was used in (A) and (B). Scale bars: 10 µm in(A-C), 5 µm in (D) and (E).

Our RT-PCR results indicated that both *Fchsd1* and *Fchsd2* mRNAs are expressed in inner ear, so we then examined the expression of the two corresponding proteins in auditory sensory epithelia using whole-mount immunostaining. The specificity of antibodies was confirmed by Western blot of native mouse inner ear tissues (Fig. 2B). In the organ of Corti, FCHSD1 immunoreactivity was detected mainly in the cuticular plate (Fig. 3A–B and Fig. S1). FCHSD2 immunoreactivity was identified in the stereocilia (Fig. 3C–E). In stereocilia, FCHSD2 immunoreactivity was distributed along the whole shaft in a punctuate pattern, where it was visible as two parallel rows flanking the F-actin core (Fig. 3D and Fig. S2).

### FCHSD2 Regulates WASP-Arp2/3-mediated F-actin Polymerization

**Figure 4:**
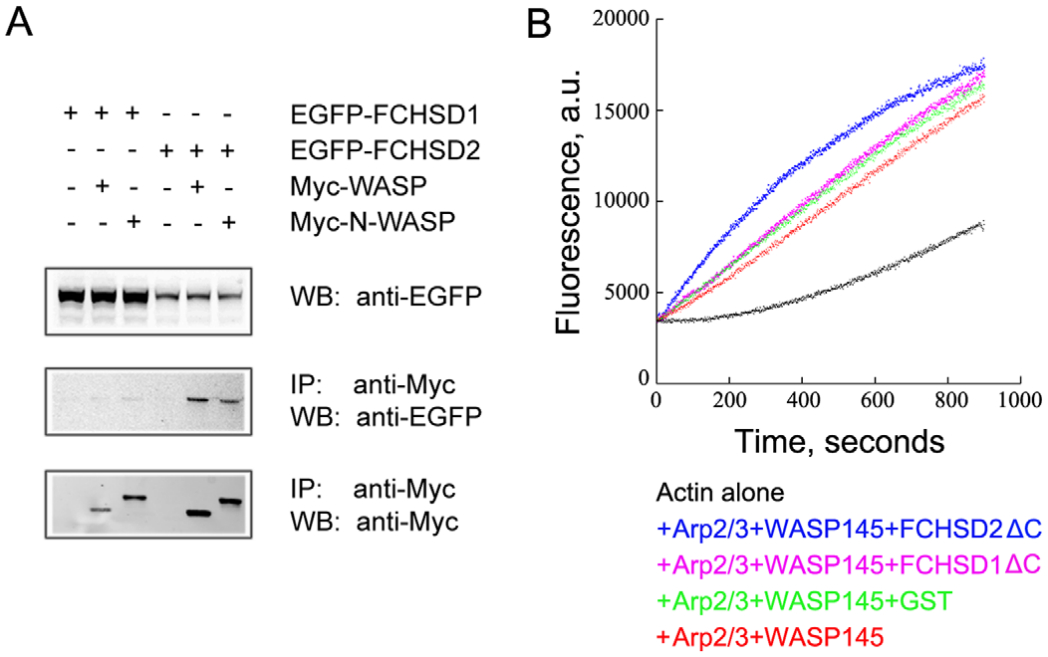
FCHSD2, but not FCHSD1, binds to WASP and N-WASP and regulates WASP-Arp2/3-mediated F-actin polymerization *in vitro*. (A) Western blots showing that FCHSD2, but not FCHSD1, was co-immunoprecipitated with WASP and N-WASP. Expression vectors were transfected into HEK293 cells to express epitope-tagged proteins, and cell lysates were subjected to immunoprecipitation. IP indicates antibody used for immunoprecipitation and WB indicates antibody used for detection. (B) WASP-Arp2/3-mediated F-actin polymerization was enhanced by GST-FCHSD2ΔC, but not by GST-FCHSD1ΔC (or GST). Monomeric actin (3 µM, 10% pyrene-labeled) was incubated with 1 µM GST-FCHSD1ΔC (or GST-FCHSD2ΔC, or GST as control) in the presence of 40 nM His-WASP145 and 20 nM Arp2/3 protein complex, and F-actin polymerization was tracked by monitoring the increase of pyrene fluorescence using a spectrofluorometer. a.u., absorbance units.

The localization of FCHSD1 and FCHSD2 in the stereocilia and the cuticular plate suggest that they might interact with F-actin in these actin-rich organelles. Because *Drosophila* Nwk has been shown to bind Wsp and regulate F-actin assembly *in vitro* [23], we hypothesized that mammalian Nwk orthologs (FCHSD1, FCHSD2) and Wsp orthologs (WASP, N-WASP) would also be able to interact in a similar fashion. To test this possibility, we expressed epitope-tagged FCHSD1, FCHSD2, WASP, and N-WASP in cultured cells, and performed co-IP experiments. Our results revealed that FCHSD2 co-immunoprecipitates with WASP and N-WASP, whereas FCHSD1 barely does so (Fig. 4A).

We then expressed FCHSD1 and FCHSD2 in *E. coli* and examined their ability to regulate F-actin polymerization *in vitro*. During the experiment we found that full-length FCHSD1 and FCHSD2 proteins accumulate in inclusion bodies in *E. coli*, which would have required refolding of the proteins for functional assays. Inclusion body formation did not happen, however, when we expressed FCHSD1ΔC and FCHSD2ΔC, which lack the carboxyl-terminal 61 and 118 amino acids, respectively, but retain F-BAR and both SH3 domains. A similar truncated Nwk protein has been shown to rescue the phenotype caused by *nwk* gene mutation in *Drosophila*, suggesting that the deleted part does not affect the function of Nwk [23]. For WASP, we expressed and purified the WASP145 fragment, which lacks the amino-terminal WH1 domain and retains its actin polymerization promoting activity [23], [33]. Our results showed that FCHSD2ΔC stimulated actin polymerization when WASP145 and Arp2/3 protein complex were present, whereas FCHSD1ΔC did not promote increased actin polymerization (Fig. 4B, compare the effect of FCHSD1ΔC with that of GST). This result is consistent with the observation that there was no detectable interaction between FCHSD1 and WASP (Fig. 4A).

### FCHSD1 Requires SNX9 for Activation of WASP-Arp2/3-mediated F-actin Polymerization

FCHSD1 does not bind WASP or N-WASP in our co-IP experiments and is unable to modulate F-actin assembly, which raised the question whether FCHSD1 can regulate F-actin assembly through other mechanisms, perhaps involving other proteins. Hence, we performed yeast two-hybrid screens of a chicken cochlear (basilar papilla) cDNA library to identify potential FCHSD1-interaction partners. Consistent with the co-IP results, neither WASP nor N-WASP was identified in the screen. Instead, we noticed a resilient interaction of the bait with a cDNA library clone encoding Sorting Nexin 9 (SNX9), a ubiquitously expressed BAR protein family member with a SH3 domain and a phox homology (PX) domain [34]. SNX9 has been shown to associate with WASP, N-WASP, Dynamin1, Dynamin2, Ack, or EspF through its SH3 domain, and functionally, it has been implicated in intracellular trafficking and F-actin assembly [27].

**Figure 5:**
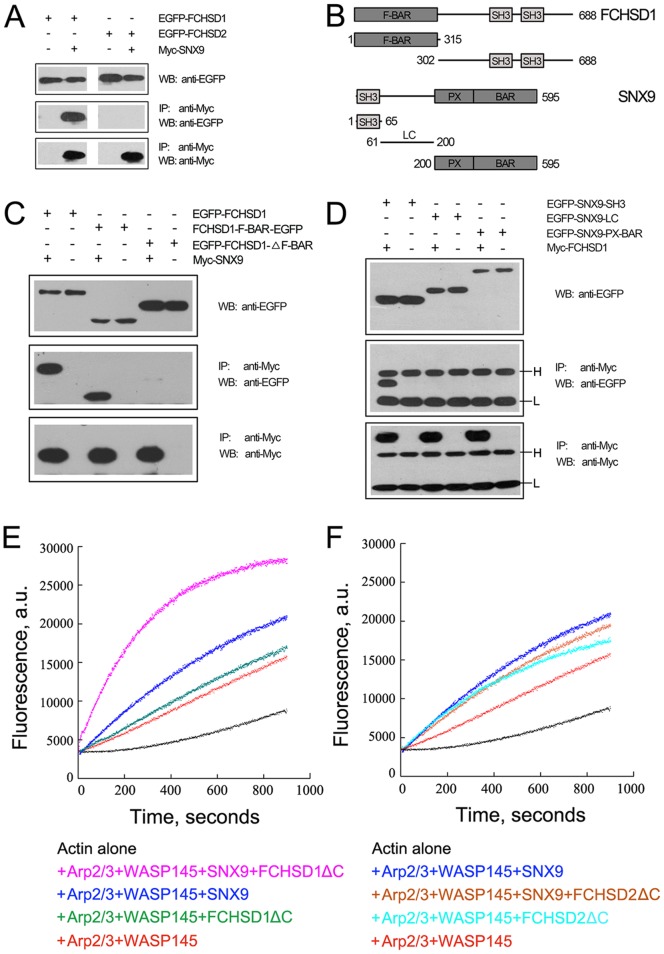
FCHSD1, but not FCHSD2, binds to SNX9 and enhances SNX9’s activity in stimulating WASP-Arp2/3-mediated F-actin polymerization *in vitro*. (A) Western blots showing that FCHSD1, but not FCHSD2, was co-immunoprecipitated with SNX9. (B) Schematic drawing of the domain structures of FCHSD1 and SNX9 that are used in the co-immunoprecipitation experiments in (C) and (D). (C) Western blots showing that the F-BAR domain of FCHSD1 was co-immunoprecipitated with SNX9. (D) Western blots showing that the SH3 domain of SNX9 was co-immunoprecipitated with FCHSD1. Expression vectors were transfected into HEK293 cells to express epitope-tagged proteins, and cell lysates were subjected to immunoprecipitation. IP indicates antibody used for immunoprecipitation and WB indicates antibody used for detection. The immunoglobulin heavy and light chains are indicated as “H” and “L”, respectively. (E) GST-SNX9 stimulated WASP-Arp2/3-mediated F-actin polymerization *in vitro*, which was enhanced by GST-FCHSD1ΔC. (F) Both GST-SNX9 and GST-FCHSD2ΔC stimulated WASP-Arp2/3-mediated F-actin polymerization *in vitro*, but there was no synergistic effect between these two proteins. Monomeric actin (3 µM, 10% pyrene-labeled) was incubated with 1 µM GST-FCHSD1ΔC (or GST-FCHSD2ΔC, or GST-SNX9) in the presence of 40 nM His-WASP145 and 20 nM Arp2/3 protein complex, and F-actin polymerization was tracked by monitoring the increase of in pyrene fluorescence using a spectrofluorometer. a.u., absorbance units.

As a confirmation assay for the yeast two-hybrid screen, we conducted co-IP experiments and found that FCHSD1 co-immunoprecipitates with SNX9, but FCHSD2 does not (Fig. 5A). We then mapped the domains responsible for the interaction between FCHSD1 and SNX9 by expressing different domains in cultured cells and repeating the co-IP experiments (Fig. 5B). We found that the F-BAR domain of FCHSD1 and the SH3 domain of SNX9 are responsible for the interaction (Fig. 5C and 5D).

The identification of SNX9 as FCHSD1 binding partner suggests that these two proteins might be able to work together to regulate F-actin assembly. It has been shown previously that SNX9 stimulates F-actin polymerization in a WASP-Arp2/3-dependent way [35]. We found that although FCHSD1ΔC had no effect on F-actin polymerization by itself, it greatly enhanced SNX9’s activity (Fig. 5E). In contrast, although both FCHSD2ΔC and SNX9 do stimulate F-actin polymerization, there is no synergistic effect between them (Fig. 5F), which is consistent with the observation that there was no detectable interaction between FCHSD2 and SNX9 (Fig. 5A).

### SNX9 Is Detectable in the Cuticular Plate

**Figure 6:**
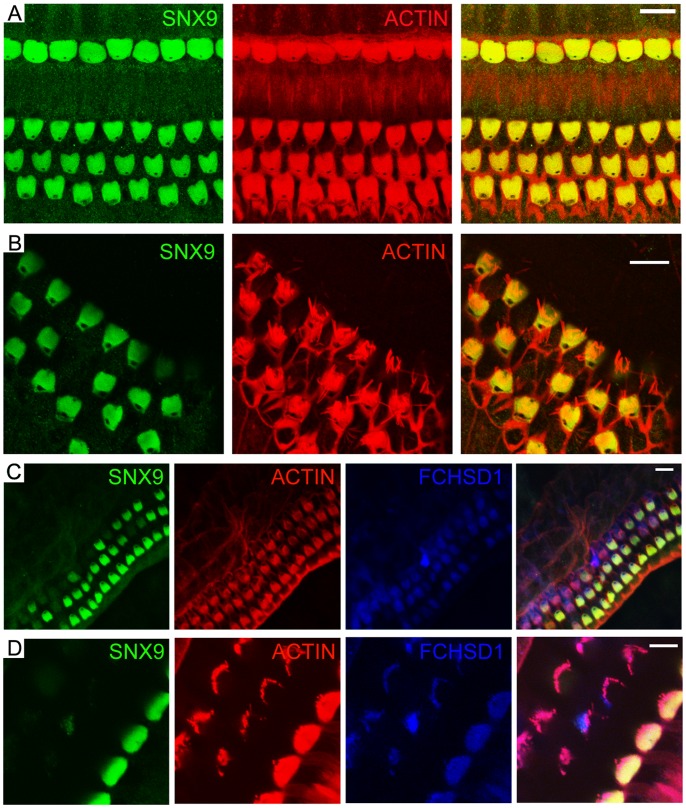
SNX9 colocalizes with FCHSD1 in the cuticular plate of mouse cochlear hair cells. Shown are single confocal sections. SNX9 immunoreactivity visualized with FITC-conjugated secondary antibody was distinctly associated with the cuticular plate, which was visualized with rhodamine-conjugated phalloidin. (A) SNX9 immunoreactivity in the cuticular plate of 2-week old mouse cochlear hair cells. (B) SNX9 immunoreactivity in the cuticular plate of 3-week old mouse outer hair cells. (C) and (D) SNX9 immunoreactivity colocalized with FCHSD1 immunoreactivity, which was visualized with Cy5-conjugated secondary antibody, in the cuticular plate of 3 week-old mouse cochlear hair cells. The Novus FCHSD1 antibody was used in (C) and (D). Scale bars: 10 µm in (A–C), 5 µm in (D).

The interaction of FCHSD1 with SNX9 raised the question whether SNX9 protein can be detected in auditory hair cells. We examined the expression of SNX9 in hair cells by performing whole-mount immunostaining with an affinity-purified polyclonal antibody against SNX9, whose specificity was confirmed by Western blot of native mouse inner ear tissues (Fig. 2B). The results showed that SNX9 immunoreactivity was clearly visible in the cuticular plate, but not in the stereocilia (Fig. 6A and 6B). In the cuticular plate, SNX9 and FCHSD1 immunoreactivity colocalized in the same confocal sections (Fig. 6C and 6D), suggesting that SNX9 and FCHSD1 might associate with each other in the cuticular plate.

## Discussion

In this study, we show that F-BAR proteins FCHSD1 and FCHSD2 localize in hair cell stereocilia and cuticular plate, and might modulate F-actin assembly or maintenance in these actin-rich organelles. Our study revealed that FCHSD1 and FCHSD2, despite the high homology of the two proteins, possess subtle but significant functional and expression differences. The most significant difference between FCHSD1 and FCHSD2 involves the ability of regulating F-actin polymerization. Similar to Nwk, FCHSD2 binds to WASP or N-WASP, and stimulates actin polymerization in a WASP-Arp2/3-dependent way. In contrast, FCHSD1 does not bind to WASP or N-WASP, and has no effect on actin polymerization when WASP and Arp2/3 are present. Nevertheless, FCHSD1 promotes F-actin polymerization via binding to SNX9. SNX9 has been shown to stimulate F-actin polymerization in a WASP-Arp2/3-dependent way [35], and FCHSD1 can bind to SNX9 and substantially enhance its activity in promoting F-actin polymerization. Another significant difference lies in their expression patterns. Notwithstanding the wide expression profiles, FCHSD1 and FCHSD2 proteins show quite distinct localizations when investigated in sensory hair cells of the cochlea. FCHSD2 mainly localizes along the stereocilia, and FCHSD1 mainly localizes to the cuticular plate, where it colocalizes with SNX9. The different localizations of FCHSD1 and FCHSD2 suggest that, although these two proteins have the ability to bind to each other, they may play distinct roles in hair cells independently of each other.

The F-actin filled stereocilia play an important role in mechanoelectrical transduction and its growth, organization, and maintenance is regulated by (F-)actin modulating proteins [36]. FCHSD2 localizes along the stereocilia, and for each stereocilium, in longitudinal section, there are two parallel rows of FCHSD2 immunoreactivity flanking the F-actin core, suggesting that the stereociliary actin core is surrounded by FCHSD2. FCHSD2 has been shown to bind MAGI-1 and CASK [26], both of which are scaffolding proteins and localize in the stereocilia [28], [37]. The immunostaining pattern of FCHSD2, however, is different from that of MAGI-1 or CASK, suggesting that these proteins may act independently in stereocilia. FCHSD2 could stimulate WASP-Arp2/3-mediated F-actin polymerization *in vitro*, but given the fact that stereocilia are composed of long, unbranched F-actin, and that Arp2/3 mainly mediate branched F-actin polymerization [38], [39], it seems unlikely that FCHSD2 plays a role in regulating actin polymerization in stereocilia. F-BAR proteins have been shown to mediate interactions between plasma membrane and cytoskeleton [21]. The localization of FCHSD2 suggests that it may play a similar role in stereocilia, providing a linker between the F-actin core and the plasma membrane.

Besides stereocilia, the cuticular plate is another actin-rich structure in hair cells. As a dense filamentous actin network, the cuticular plate occupies the apical cell cytoplasm of hair cells and serves as an anchor for stereocilia [2], [3]. Different from stereocilia, the cuticular plate is mainly composed of highly branched actin filaments. Several proteins have been shown to localize in the cuticular plate, including alpha-actinin, spectrin, fimbrin, protocadherin 15, myosin 6, and myosin 7a [40], [41], [42], [43], [44], [45], [46], [47], [48], [49]. Mutations in *protocadherin 15*, *myosin 6*, or *myosin 7a* genes can cause disorganization of cuticular plate or result in stereocila fusion [50], [51], [52], [53]. But the mechanism about how the development and maintenance of this actin-rich organelle is regulated remains to be elucidated. Here we show that FCHSD1 mainly localizes to the cuticular plate in hair cells. More interestingly, the FCHSD1-binding protein SNX9, although ubiquitously expressed in almost all the tissues examined [34], is only detected in the cuticular plate within hair cells. The localization of these two proteins in this actin-rich organelle, combined with the fact that they stimulate F-actin polymerization *in vitro*, suggest that FCHSD1 and SNX9 might work together and regulate actin polymerization in the cuticular plate. To our knowledge, this is the first report of actin-regulatory proteins expressed in the cuticular plate. At present, the expression of Arp2/3, WASP, or N-WASP in hair cells is not known. The knowledge of expression patterns of these proteins in hair cells, especially in cuticular plate, will help us to learn more about how FCHSD1 and SNX9 regulate actin assembly in this actin-rich organelle.

In summary, we report here that FCHSD1 and FCHSD2 are two actin-regulatory proteins that localize in cochlear hair cell stereocilia and cuticular plate. Their different localizations suggest that these two proteins could play distinctive roles, such as maintaining the stability and integrity, or regulating F-actin dynamics, of these actin-rich organelles. Our data show that FCHSD1 and FCHSD2 accelerate F-actin polymerization in direct cooperation with WASP-Arp2/3 or indirectly, via SNX9. Although not detectable in stereocilia, SNX9 was detected in the cuticular plate, where it colocalizes with FCHSD1. The localization of FCHSD1 and SNX9 in cuticular plate and their ability to regulate F-actin polymerization could be an indication for a functional role in maintaining the stability and/or regulating the F-actin turnover in the cuticular plate. Further investigations need to be done to fully understand how these proteins are involved in maintaining and/or regulating actin filaments in hair cell stereocilia and cuticular plate. At present, knockout mice of FCHSD1, FCHSD2, or SNX9 are not available; more knowledge will be obtained when these knockout mice are developed and analyzed.

## Supporting Information

Figure S1 **FCHSD1 immunolocalizaiton in mouse cochlear hair cells.** (A) Total proteins of postnatal day 5 mouse cochlea and vestibula were extracted and separated by PAGE and detected with anti-FCHSD1 antibody. (B) FCHSD1 immunoreactivity in the cuticular plate of 10-week old mouse cochlear hair cells. (C) FCHSD1 immunoreactivity in the cuticular plate of 4-week old mouse cochlear hair cells. The Abcam FCHSD1 antibody was used in (A–C). Scale bars: 5 µm.
(TIF)

Figure S2 **FCHSD2 immunolocalization in mouse cochlear inner hair cell stereocilia.** Shown are single confocal sections enlarged from Figure 3D. FCHSD2 immunoreactivity visualized with FITC-conjugated secondary antibody was distributed along the whole shaft in a punctuate pattern, where it was visible as two parallel rows flanking the F-actin core, which was visualized with rhodamine-conjugated phalloidin. Scale bar: 1 µm.
(TIF)

## Acknowledgments

The authors thank Dr. Shanjin Huang (Institute of Botany, Chinese Academy of Sciences) for advice and help with the actin polymerization assay, and Dr. Yutaka Hata (Department of Medical Biochemistry, Graduate School of Medicine, Tokyo Medical and Dental University) for providing the antibody against FCHSD2, as well as Dr. Michael Rosen (UT Southwestern Medical Center) for providing WASP and N-WASP cDNAs.
