# Supplementary figures and images for: FCHSD1 and FCHSD2 Are Expressed in Hair Cell Stereocilia and Cuticular Plate and Regulate Actin Polymerization In Vitro
Source: PLoS One. 2013 Feb 20;8(2):e56516. doi: 10.1371/journal.pone.0056516 (PMC3577914; doi:10.1371/journal.pone.0056516)

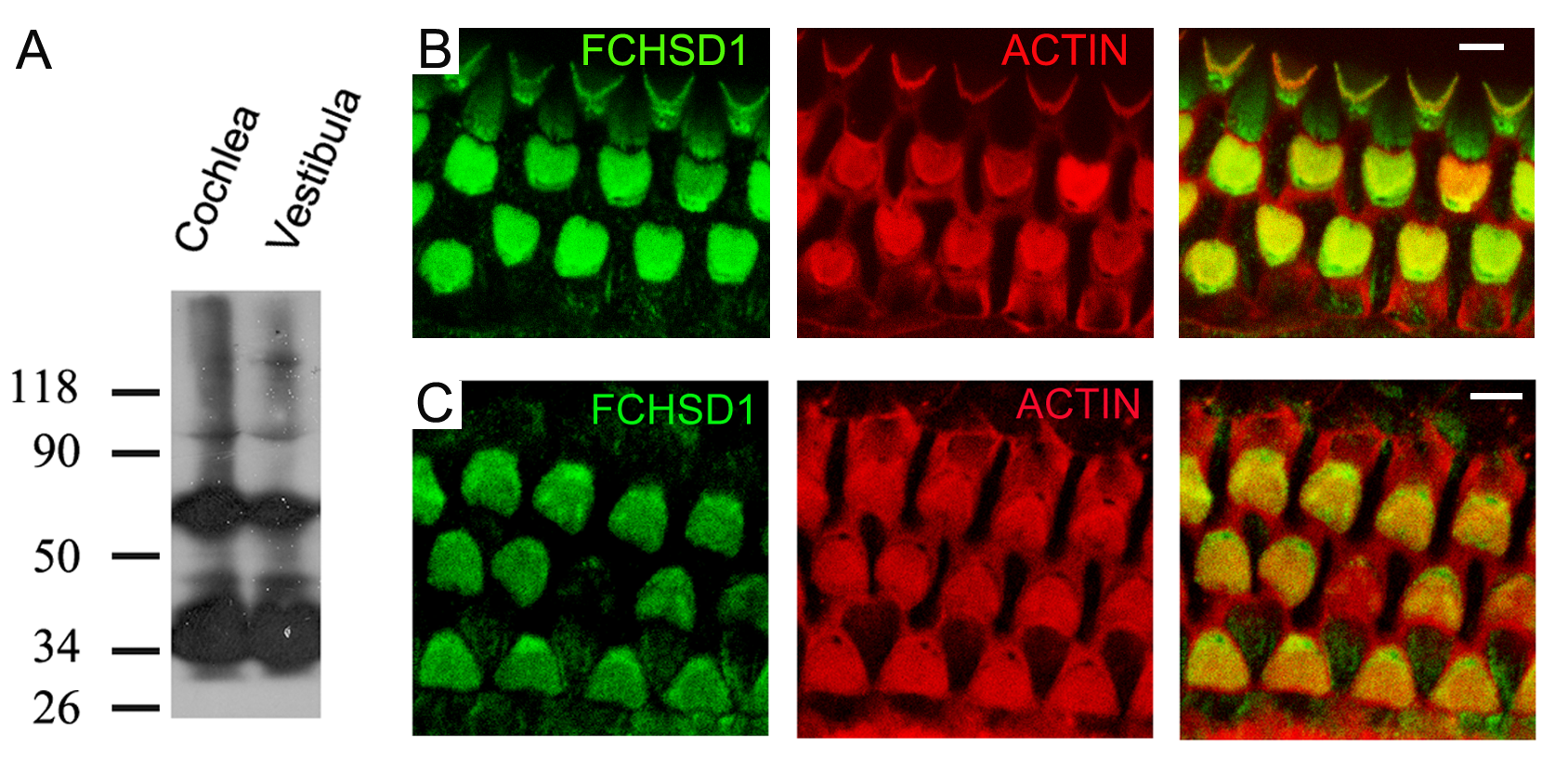

Supplement: Figure S1 — FCHSD1 immunolocalizaiton in mouse cochlear hair cells. (A) Total proteins of postnatal day 5 mouse cochlea and vestibula were extracted and separated by PAGE and detected with anti-FCHSD1 antibody. (B) FCHSD1 immunoreactivity in the cuticular plate of 10-week old mouse cochlear hair cells. (C) FCHSD1 immunoreactivity in the cuticular plate of 4-week old mouse cochlear hair cells. The Abcam FCHSD1 antibody was used in (A–C). Scale bars: 5 µm. (TIF) [file pone.0056516.s001.tif]

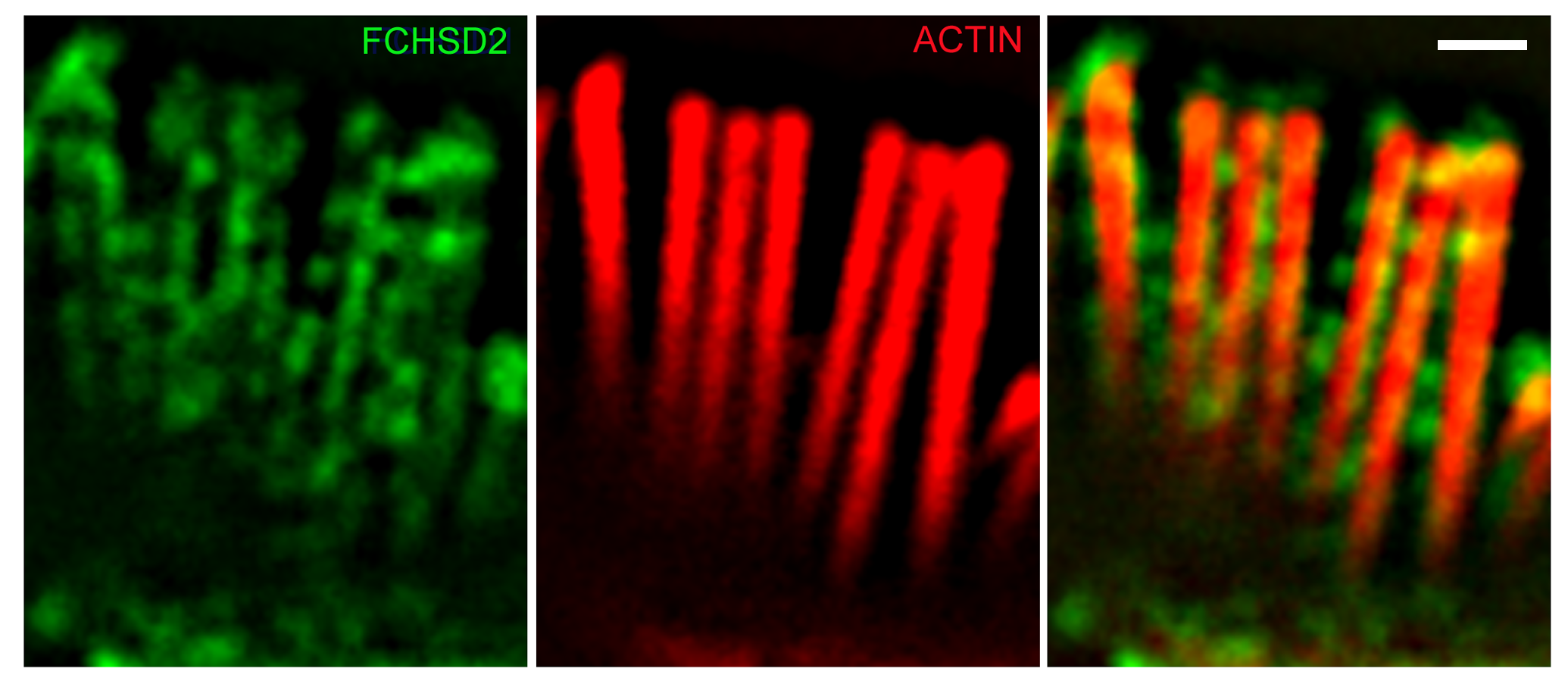

Supplement: Figure S2 — FCHSD2 immunolocalization in mouse cochlear inner hair cell stereocilia. Shown are single confocal sections enlarged from Figure 3D. FCHSD2 immunoreactivity visualized with FITC-conjugated secondary antibody was distributed along the whole shaft in a punctuate pattern, where it was visible as two parallel rows flanking the F-actin core, which was visualized with rhodamine-conjugated phalloidin. Scale bar: 1 µm. (TIF) [file pone.0056516.s002.tif]
